# Real-World Effectiveness of Inhalation Therapy Among Patients With Symptomatic COPD in China: A Multicenter Prospective Study

**DOI:** 10.3389/fphar.2021.753653

**Published:** 2021-09-21

**Authors:** Wei Cheng, Jiaxi Duan, Aiyuan Zhou, Yiyang Zhao, Rong Yi, Yi Liu, Dingding Deng, Xin Li, Yuqin Zeng, Yating Peng, Qing Song, Ling Lin, Min Yang, Ping Chen

**Affiliations:** ^1^ Department of Pulmonary and Critical Care Medicine, Research Unit of Respiratory Disease, Diagnosis and Treatment Center of Respiratory Disease, The Second Xiangya Hospital, Central South University, Changsha, China; ^2^ Department of Pulmonary and Critical Care Medicine, Xiangya Hospital, Central South University, Changsha, China; ^3^ Department of Pulmonary and Critical Care Medicine, Zhuzhou Central Hospital, Zhuzhou, China; ^4^ Department of Respiratory Medicine, The First Affiliated People’s Hospital, Shaoyang College, Shaoyang, China; ^5^ Division 4 of Occupational Diseases, Hunan Prevention and Treatment Institute for Occupational Diseases, Changsha, China

**Keywords:** COPD-chronic obstructive pulmonary disease, symptomatic, inhalation therapy, real-world, exacerbation, MCID (minimal clinically important differences), COPD assessment test (CAT)

## Abstract

**Purpose:** This real-world study evaluated the effectiveness of different inhalation therapies in patients with symptomatic chronic obstructive pulmonary disease (COPD) in China and also explored the relevant factors that influence the effectiveness of inhalation therapy.

**Patients and Methods:** We conducted a multicenter prospective longitudinal study that was carried out in 12 hospitals in China from December 2016 to June 2021. A face-to-face interview was conducted to collect data. Baseline data were collected at the first visit. Minimum clinically important difference (MCID) was defined as attaining a COPD assessment test (CAT) decrease ≥2. We mainly assessed the MCID and the incidence of exacerbations at the 6 months follow-up.

**Results:** In 695 patients, the mean age was 62.5 ± 8.2 years, with a mean CAT score of 15.1 ± 6.0. Overall, 341 (49.1%) patients attained the MCID of CAT and the incidence of exacerbation during follow-up was 22.3%. Females were significantly more likely to attain MCID than male in COPD patients (adjusted odd ratio (aOR) = 1.93, adjusted 95% confidence interval (a95%CI) = 1.09–3.42, *p* = 0.024). Patients treated with LABA/LAMA or ICS/LABA/LAMA (ICS, inhaled corticosteroid; LABA, long-acting β2-agonist; LAMA, long-acting muscarinic antagonist) were more likely to attain MCID than patients treated with LAMA (aOR = 3.97, a95%CI = 2.48–6.35, *p* < 0.001; aOR = 3.17, a95%CI = 2.09–4.80, *p* < 0.001, respectively). Patients treated with LABA/LAMA had a higher incidence of severe exacerbation than patients treated with ICS/LABA/LAMA (aOR = 1.95, a95%CI = 1.04–3.66, *p* = 0.038).

**Conclusion:** The incidence of MCID in symptomatic COPD patients treated with inhalation therapy was nearly 50%. Patients treated with LABA/LAMA or ICS/LABA/LAMA were more likely to attain MCID than patients treated with LAMA. Patients treated with LABA/LAMA had a higher incidence of severe exacerbations than with ICS/LABA/LAMA.

## Introduction

Chronic obstructive pulmonary disease (COPD) is a chronic respiratory disease with persistent airflow limitation caused by toxic particles or gases (Vogelmeier et al., 2017). Globally, 174.5 million (2.4%) people suffer from COPD (GBD 2015 Disease and Injury Incidence and Prevalence Collaborators, 2016), and the prevalence in patients over 40 years of age in China is 13.7% (Wang et al., 2018). COPD is now one of the top three causes of death worldwide (Lozano et al., 2012).

With the progression of COPD, the burden of symptoms increases and quality of life declines. Symptomatic patients with COPD (group B and D) account for the vast majority in China (Duan et al., 2020). Furthermore, compared with patients with well-controlled symptoms, more symptomatic patients have a higher risk of acute exacerbations and poorer disease prognosis (Roche et al., 2013; Miravitlles and Ribera, 2017). Thus, we need to pay more attention to this group so as to further optimize the management of patients with symptomatic COPD.

At present, the effectiveness of different inhaled bronchodilators (long-acting muscarinic antagonist (LAMA); inhaled corticosteroids (ICS)/long-acting β_2_-agonists (LABA); as well as the combinations LABA/LAMA and ICS/LABA/LAMA) in the treatment of COPD patients is still controversial (Wedzicha et al., 2016; Lipson et al., 2018; Maltais et al., 2019; Suissa et al., 2019; Lipson et al., 2020; Rabe et al., 2020). These therapies have been tested in randomized controlled trials (RCTs) with strict inclusion and exclusion criteria. The effectiveness of treatment evaluated in real-world studies can complement traditional RCTs by providing a comprehensive overview of treatments in routine clinical practice. Previous real-world studies usually selected one or two types of bronchodilators in mono, dual combination or triple combinations for analysis (Kalhan et al., 2021; Sansbury et al., 2021; Xu et al., 2021), and some studies have compared the effect between open triple and closed triple therapy (Ferguson et al., 2020; Huang et al., 2021). However, there is a lack of real-world data on the effects of the inhalation therapies including mono, dual combination and triple combination therapies among patients with symptomatic COPD in China.

Therefore, the purpose of this real-world study was to compare the effectiveness of different inhalation therapies for symptomatic COPD patients in China and to explore the relevant factors that influence the effectiveness of inhalation therapy.

## Materials and Methods

### Study Participants and Procedures

We conducted a multicenter prospective longitudinal cohort study that was carried out in 12 comprehensive hospitals (Supplementary Table S1) in China from December 2016 to June 2021. We collected data by conducting face-to-face interviews with patients. All study participants provided signed informed consent. The baseline data of all participants were collected at the first visit. At the first visit of 695 patients at these centers, 624 (89.8%) patients received inhalation treatment for the first time, and 71 (10.2%) patients received adjusted treatment including 26 patients adjusted from LAMA to LABA/LAMA, two patients adjusted from LAMA to ICS/LABA/LAMA, 16 patients adjusted from ICS/LABA to LABA/LAMA, two patients adjusted ICS/LABA to ICS/LABA/LAMA, and 25 patients adjusted from ICS/LABA/LAMA to LABA/LAMA.

We confirmed that this research was conducted in accordance with the Declaration of Helsinki and has been registered in the Chinese Clinical Trial Registry (ChiCTR-POC-17010431). The study protocol was approved by the local Ethics Committee of the Second Xiangya Hospital of Central South University.

The inclusion criteria for patients in this study were that they: 1) met the diagnosis criterion of COPD defined by the 2017 Global Initiative for Chronic Obstructive Lung Disease (GOLD) recommendations [spirometry with a ratio of the forced expiratory volume in 1 s to the forced vital capacity (FEV1/FVC) lower than 0.70 after bronchodilator administration] (Vogelmeier et al., 2017); 2) were over 40 years of age; 3) a score on the COPD Assessment Test (CAT) ≥10 and or mMRC ≥2. Exclusion criteria were: 1) patients with acute exacerbation of COPD (AECOPD, an acute worsening of respiratory symptoms that results in additional therapy in patients with COPD (Vogelmeier et al., 2017); 2) patients with other chronic respiratory diseases, such as bronchiectasis (based on high-resolution computed tomography), asthma (clinically diagnosed and reversibility >12%), interstitial lung disease, or concurrent malignancy (including lung cancer); 3) patients with severe heart, liver, or kidney diseases (based on actual diagnoses from case records).

### Baseline Demographics and Clinical Characteristics

Baseline characteristics included age at index date, sex, body height (BH), body weight (BW), body mass index (BMI), and smoking status. A smoker was defined as continuous smoking exposure of more than 10 pack-years. Patients who had abstained for more than 6 months were classified as former smokers (Liu et al., 2020). Never smokers were defined as those with a lifetime exposure of <1/20 pack-year (Tan et al., 2015). Clinical characteristics of interest were pulmonary function tests, CAT score, Modified Medical Research Council Dyspnea Scale (mMRC) score, number of previous exacerbations at baseline, severity of exacerbation (moderate or severe), smoking history, occupational exposure or biofuel exposure history, the presence of comorbidities ever recorded, and inhalation therapy drugs.

COPD disease severity was classified using the GOLD guidelines and was divided into four stages: mild (FEV1 ≥80% predicted), moderate (FEV1 50–80% predicted), severe (FEV1 30–50% predicted), or very severe (FEV1 <30% predicted). Dyspnea was measured by using the mMRC. The COPD assessment test (CAT) consists of eight items, including cough, expectoration, dyspnea, chest tightness, confidence, limitation of daily activities, quality of sleep, and levels of energy with a total scores ranging from 0 to 40. Our study only investigated moderate and severe exacerbations in the previous year and during the follow-up. Moderate exacerbations were defined as those requiring a prescription for an oral corticosteroid and/or an antibiotic on the same date, and severe exacerbation required an emergency department attendance or a hospital admission (Vogelmeier et al., 2017). The GOLD BD groups (symptomatic COPD) were defined according to the patient’s symptoms and the history of exacerbations in the past 1 year as follows: Group B: 0–1 exacerbations per year, no hospitalization, mMRC ≥2 and or CAT ≥10; Group D: ≥2 exacerbations per year, ≥1 exacerbation with hospitalization, mMRC ≥2 and or CAT ≥10.

### Treatment Assessment

We evaluated the effectiveness of inhalation therapy based on the response rate of the minimum clinically important difference (MCID) of CAT during the 6 months follow-up. MCID, defined as attaining minimum clinically important difference of CAT (decrease ≥2) (Kon et al., 2014), was assessed at 6 months follow-up. Response rates were calculated based on the proportion of individual patients with a ≥2-unit improvement in CAT score from baseline. We also assessed the incidence of moderate/severe acute exacerbations (AEs) and prescription outcome during the 6 months follow-up.

Adherence was calculated using the medication possession ratio (MPR). MPR was calculated by summing the days of medication supply provided and dividing by the total time treated (Covvey et al., 2014). Patients with poor adherence (MPR <80% or MPR >120%) were not included in the evaluation of effectiveness during the 6 months follow-up. Five mutually exclusive prescription outcomes were defined: continuous use (no modification), discontinuation (permanent [≥91 days with no restart] or temporary [≥91 days with subsequent restart]), switch, and augmentation (Meeraus et al., 2019). Participants who received escalation long-acting bronchodilator therapy or augmented long-acting bronchodilator therapy before the 6 months follow-up, regardless of whether they met the above requirements, were classified as non-MCID.

### Sample Size Estimation

The sample size was calculated by using PASS 15.0 in the part of confidence intervals for one proportion. We used the MCID incidence rate (44.9%) obtained from the pre-experiment as the assumed sample proportion, set the interval type as two-sided, and entered the confidence level (1-alpha) as 0.95 and dropout rate as 10%. Finally, the sample we acquired was 679.

### Statistical Analysis

Categorical variables are described as counts and percentages. Continuous variables are expressed as mean ± standard deviation or median with interquartile range (IQR) according to normally or non-normally distributed. The chi-squared or Fisher’s test was used for categorical variables, and Student’s t-test, Mann-Whitney U test, and Kruskal-Wallis H test were used for continuous variables. Risk factors for MCID of CAT and severe exacerbation during follow-up were identified, and their crude odds ratios (cORs), adjusted odds ratios (aORs), and 95% confidence intervals were estimated using logistic regression analyses. All tests of significance were two sided, and a *p* value < 0.05 was considered to be statistically significant. Multiple comparisons of differences between groups were Bonferroni adjusted. All analyses were performed using IBM SPSS Statistics version 25.0 for Windows (IBM Corp, Armonk, NY, United States).

## Results

### Baseline Characteristics

**FIGURE 1:**
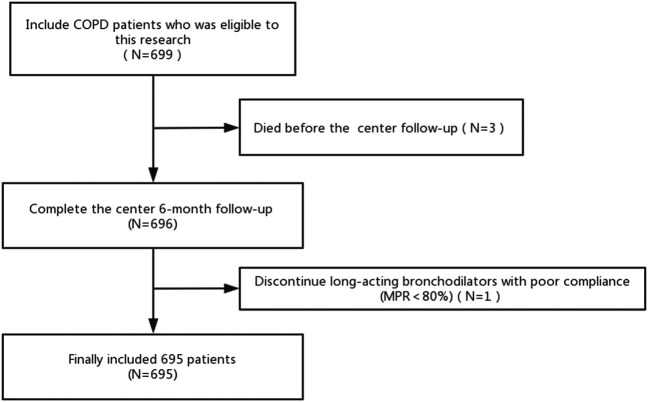
Flow diagram of the inclusion of studies. Abbreviations: COPD, chronic obstructive pulmonary disease; MPR, medication possession ratio.

A total of 696 patients completed the center 6 months follow-up. One patient discontinued long-acting bronchodilators with poor compliance (MPR<80%). Finally, we included 695 patients for analysis (Figure 1).

Of the 695 patients in the baseline, 90.6% were male, with a mean age of 62.5 ± 8.2 years, a mean CAT score of 15.1 ± 6.0, a median FEV1 percentage predicted of 48.3 ± 25.5% and a median FEV1 of 1.21 ± 0.54. These COPD patients included 344 patients in group B and 351 patients in group D. The distribution of inhalation therapy was as follows: LAMA (24.3%), LAMA/LABA (21.4%), ICS/LABA (10.4%), ICS/LABA/LAMA (35.3%), Others (8.6%) including ICS/LAMA and short-acting bronchodilators. Baseline demographics and clinical characteristics are summarized in Table 1.

**TABLE 1:** Baseline demographics and clinical characteristics.

Baseline characteristics	Total group (*N* = 695)
Age^a^ (year)	62.5 (8.2)
BMI^a^ (kg/m^2^)	22.3 (3.1)
FEV_1_ ^c^ (liter)	1.21 (0.54)
FEV_1_% predicted^c^ (%)	48.3 (25.5)
CAT^a^	15.1 (6.0)
mMRC^c^	2.0 (2.0)
Male^b^	630 (90.6)
Current smoker^b^	287 (41.3)
Occupational exposure^b^	242 (34.8)
Biofuel exposure^b^	219 (31.5)
Exacerbation in the past 1 year^b^	
0	283 (40.7)
≥1	412 (59.3)
COPD severity^b^	
Mild	40 (5.8)
Moderate	282 (40.6)
Severe	288 (41.4)
Very severe	85 (12.2)
Group B/D^b^	
B	344 (49.5)
D	351 (50.5)
Inhalation^b^	
LAMA	169 (24.3)
LAMA/LABA	149 (21.4)
ICS/LABA	72 (10.4)
ICS/LABA/LAMA	245 (35.3)
Others	60 (8.6)

a Mean (SD).

b Counts with percentage are indicated.

c Median (IQR).

**Abbreviations:** BMI, body mass index; FEV1%, forced expiratory volume in one second as a percentage of the predicted value; CAT, COPD assessment test; mMRC, modified medical research council dyspnea scale; COPD severity was classified using Global Initiative for Chronic Obstructive Lung Disease (GOLD) criteria; LABA, long-acting β2-agonist; LAMA, long-acting muscarinic antagonist; ICS, inhaled corticosteroid.

As shown in Supplementary Table S2, the proportion of patient with a history of exacerbation in the past year was higher in COPD patients treated with LAMA (111/169 = 65.7%) than in patients treated with LABA/LAMA (78/149 = 52.3%). Furthermore, the proportion of patients with a history of severe exacerbations in the past year was higher in COPD patients treated with LABA/LAMA (60/169 = 40.3%) and ICS/LABA/LAMA (93/245 = 38.0%) than in patients treated with ICS/LABA (16/72 = 22.2%).

### Effectiveness of Different Inhalation Therapies

**FIGURE 2:**
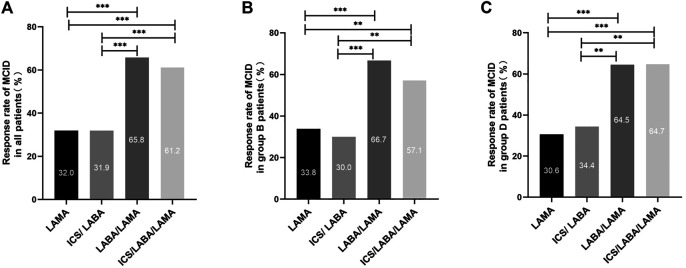
Comparison of the MCID response rate between different main inhalation therapy in patients with symptomatic COPD. Note: **(A)** MCID response rate in all patients with symptomatic COPD. **(B)** MCID response rate in group B patients with symptomatic COPD. **(C)** MCID response rate in group D patients with symptomatic COPD. For comparison, the chi-squared test was used for categorical variables. ** indicates *p*-values <0.01, *** indicates *p*-values <0.001. Abbreviations: MCID, minimum clinically important difference; LABA, long-acting β2-agonist; LAMA, long-acting muscarinic antagonist; ICS, inhaled corticosteroid.

As exhibited in Table 2, 341 (49.1%) patients attaining MCID of CAT (decrease ≥2) assessed at the 6 months follow-up. There were 275 (39.6%) patients attaining an mMRC decrease ≥1 assessed at the 6 months follow-up. In all participants, the inhalation treatment of COPD patients with LAMA/LABA (98/149 = 65.8%) or ICS/LABA/LAMA (150/245 = 61.2%) had a higher response rate regarding MCID than LAMA (54/169 = 32.0%) or ICS/LABA (23/72 = 31.9%). Regardless of group B or D, the inhalation therapy of COPD patients with LAMA/LABA or ICS/LABA/LAMA (triple therapy) had a higher response rate regarding MCID than therapy with LAMA or ICS/LABA (Figure 2).

**TABLE 2:** Effectiveness of different inhalation therapy options during 6 months follow-up.

Outcome	Total (*N* = 695)	LAMA (*N* = 169)	LAMA/LABA (*N* = 149)	ICS/LABA (*N* = 72)	ICS/LABA/LAMA (*N* = 245)	Others (*N* = 60)	*p*-value
Δ CAT, Median (IQR)	2 (8)	0 (7)	4 (8.5)	0 (7.75)	3 (9)	−0.5 (4)	<**0.001**
MCID of CAT, *n* (%)							<**0.001**
Yes	341 (49.1)	54 (32.0)	98 (65.8)	23 (31.9)	150 (61.2)	16 (26.7)	
No	354 (50.9)	115 (68.0)	51 (34.2)	49 (68.1)	95 (38.8)	44 (73.3)	
AE during 6 months follow-up, Median (IQR)	0 (1)	1 (2)	0 (0)	0 (1.75)	0 (2)	0 (2)	<**0.001**
AE during 6 months follow-up, *n* (%)							0.932
Yes	154 (22.2)	37 (21.9)	31 (20.1)	14 (19.4)	58 (23.7)	14 (23.3)	
No	541 (77.8)	132 (78.1)	118 (79.9)	58 (80.6)	187 (76.3)	46 (76.7)	
Severe AE during 6 months follow-up, *n* (%)							**0.011**
Yes	60 (8.6)	8 (4.7)	23 (15.4)	4 (5.6)	21 (8.6)	4 (6.7)	
No	635 (91.4)	161 (95.3)	126 (84.6)	68 (94.4)	224 (91.4)	56 (93.3)	
Prescription outcome, *n* (%)							<**0.001**
Continuous using	571 (82.1)	129 (76.3)	145 (97.3)	47 (65.3)	191 (78.0)	59 (98.3)	
De-escalation therapy	66 (9.5)	0 (0)	3 (2.0)	9 (12.5)	54 (22.0)	0 (0)	
Escalation therapy	6 (0.9)`	4 (2.4)	1 (0.7)	0 (0)	0 (0)	1 (1.7)	
Augmented	52 (7.5)	36 (21.3)	0 (0)	16 (22.2)	0 (0)	0 (0)	

**Note:** For comparison, Chi-square or Fisher’s test was used for categorical variables, and Kruskal-Wallis H test were used for continuous variables; the bold *p*-values indicate statistical significance.

**Abbreviations:** CAT, COPD assessment test; Δ CAT was calculated by subtracting the baseline CAT score from the follow-up CAT score; MCID, minimum clinically important difference, defined as attaining minimum clinically important differences of CAT (decrease ≥2) assessed at 6 months follow-up; AE, acute exacerbation; LABA, long-acting β2-agonist; LAMA, long-acting muscarinic antagonist; ICS, inhaled corticosteroid.

Overall, the incidence of exacerbations during follow-up was 22.25%. The incidence of exacerbations during the 6 months follow-up with different inhalation therapies was as follows: LAMA (21.9%), LAMA/LABA (20.1%), ICS/LABA (19.4%), ICS/LABA/LAMA (23.7%), others (23.3%); however, we found no difference in the rate of exacerbations between these inhalation treatments. We found that there were significant differences in the incidence of severe exacerbations among patients on different inhalation therapies during follow-up (*p* = 0.011) (Table 2). Further subgroup analysis showed that, with different inhalation therapies, patients who had a history of exacerbation in the past year exhibited a variable incidence of severe exacerbations in follow-up (*p* = 0.009), while patients without a history of exacerbation had a similar prognosis (*p* = 0.752).

### Factors Correlated With the MCID Response Rate

In Table 3, female (66.2%) COPD patients had a higher MCID response rate than males with COPD (47.3%). As shown in Figure 2, there were significant differences in the MCID response rate between different inhalation therapies (*p* < 0.01). We found no significant differences in the MCID response rate during the 6 months follow-up according to different treatment status at baseline, while patients in the LABA/LAMA subgroup had similar results (*p* = 0.158). After adjusting for sex, age, smoking status, treatment status at baseline, exacerbation history in the past year, GOLD stage, group B/D, and inhalation therapy, the logistic regression model showed that females were significantly more likely to attain MCID than male COPD patients (aOR = 1.93, a95%CI = 1.09–3.42, *p* = 0.024). We also found that patients treated with LABA/LAMA or ICS/LABA/LAMA were more likely to attain MCID than patients treated with LAMA (aOR = 3.97, a95%CI = 2.48–6.35, *p* < 0.001; aOR = 3.17, a95%CI = 2.09–4.80, *p* < 0.001, respectively) (Table 4).

**TABLE 3:** Response rate of MCID between different clinical features for symptomatic COPD patients.

Clinical feature	Total, *N*	Patients with MCID, *n* (%)	Patients without MCID, *n* (%)	*p*-value
Age (year)				0.131
<65	387	180 (46.5)	207 (53.5)	
≥65	308	161 (52.3)	147 (47.7)	
Sex				**0.004**
Male	630	298 (47.3)	332 (52.7)	
Female	65	43 (66.2)	22 (33.8)	
BMI (kg/m^2^)				0.575
<24	500	242 (48.4)	258 (51.6)	
≥24	195	99 (50.8)	96 (49.2)	
Smoking history				0.290
Never smoker	134	66 (49.3)	68 (50.7)	
Former smoker	274	125 (45.6)	149 (54.4)	
Current smoker	287	150 (52.3)	137 (47.7)	
Occupational exposure				0.218
Yes	242	111 (45.9)	131 (54.1)	
No	453	230 (50.8)	223 (49.2)	
Biofuel exposure				0.813
Yes	219	106 (48.4)	113 (51.6)	
No	476	235 (49.4)	241 (50.6)	
AE in the past 1 year				0.738
0	285	142 (49.8)	143 (50.2)	
≥1	410	199 (48.5)	211 (51.5)	
COPD severity				0.212
Mild	40	20 (50.0)	20 (50.0)	
Moderate	282	126 (44.7)	156 (55.3)	
Severe	288	147 (51.0)	141 (49.0)	
Very severe	85	48 (56.5)	37 (43.5)	
Group B/D				0.673
Group B	344	166 (48.3)	178 (51.7)	
Group D	351	175 (49.1)	176 (50.9)	
Treatment status at baseline				0.073
Initial treatment	624	299 (47.9)	325 (52.1)	
Adjust treatment	71	42 (59.2)	29 (40.8)	

**Note:** For comparison, Chi-square was used for categorical variables; the bold *p*-values indicate statistical significance.

**Abbreviations:** MCID, minimum clinically important difference; BMI, body mass index; AE, acute exacerbation; COPD severity was classified using Global Initiative for Chronic Obstructive Lung Disease (GOLD) criteria.

**TABLE 4:** Multiple logistic regression for factors correlated with the response rate of MCID.

Characteristics (*N* = 695)	cOR	c95%IC	*p*-value	aOR	a95%IC	*p*-value
Sex			**0.005**			**0.024**
male	Reference			Reference		
female	2.18	1.27–3.73		1.93	1.09–3.42	
Inhalation therapy			<**0.001**			
LAMA	Reference			Reference		
LAMA/LABA	4.09	2.56–6.54	<**0.001**	3.97	2.48–6.35	<**0.001**
ICS/LABA	1.00	0.55–1.81	0.999	0.90	0.49–1.64	0.726
ICS/LABA/LAMA	3.36	2.23–5.08	<**0.001**	3.17	2.09–4.80	<**0.001**
Others	0.77	0.40–1.49	0.446	0.78	0.41–1.51	0.462

**Note:** Factors in the logistic model: sex, age, smoking status, treatment status at baseline, exacerbation history in the past 1 year, Gold stage, group B/D, Inhalation therapy; the bold *p*-values indicate statistical significance.

**Abbreviations:** MCID, minimum clinically important difference; LABA, long-acting β2-agonist; LAMA, long-acting muscarinic antagonist; ICS, inhaled corticosteroid; cOR, crude odds ratio; c95% CI, crude 95% confidence interval; aOR, adjusted odds ratio; a95% CI, adjusted 95% confidence interval.

### Factors Correlated With the Incidence of Severe Exacerbations

**FIGURE 3:**
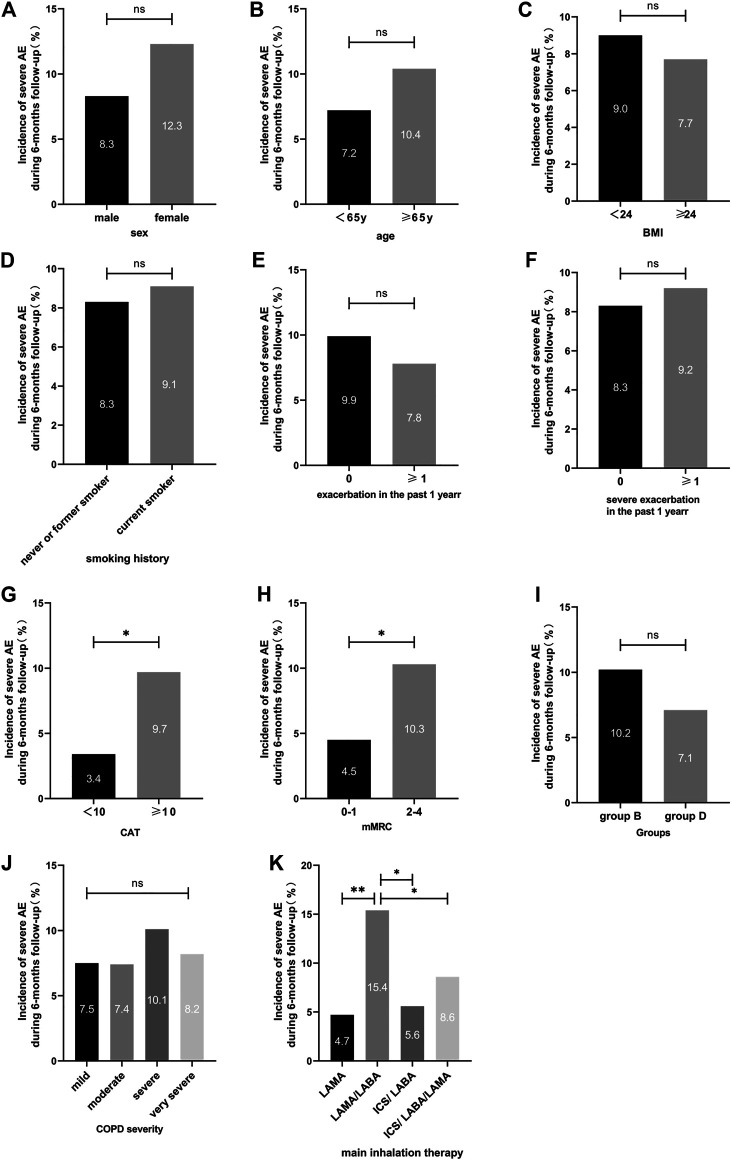
Incidence of severe exacerbations during the 6-months follow-up between different clinical features for symptomatic COPD patients. Note: For comparison, the chi-squared test was used for categorical variables. ns indicates *p*-values ≥0.05, * indicates *p*-values <0.05, ** indicates *p*-values <0.01. Abbreviations: BMI, body mass index; CAT, COPD assessment test; mMRC, modified medical research council dyspnea scale; COPD severity was classified using Global Initiative for Chronic Obstructive Lung Disease (GOLD) criteria; LABA, long-acting β2-agonist; LAMA, long-acting muscarinic antagonist; ICS, inhaled corticosteroid.

The incidence of severe exacerbations in patients was significantly related to the CAT score and the mMRC score (9.7 vs. 3.4%, *p* = 0.029; 4.5 vs. 10.3%, *p* = 0.015, respectively). Inhalation treatment of COPD patients with LAMA (8/169 = 4.7%), ICS/LABA (4/72 = 5.6%), and ICS/LABA/LAMA (21/245 = 8.6%) had a lower incidence of severe exacerbations than LABA/LAMA (23/149 = 15.4%) during the 6 months follow-up (Table 2 and Figure 3). After adjusting for sex, age, treatment status at baseline, exacerbation in the past year, severe exacerbation in the past year, CAT score, mMRC score, GOLD stage, group B/D, and inhalation therapy, the logistic regression model showed that patients treated with LABA/LAMA had a higher incidence of severe exacerbations than patients treated with ICS/LABA/LAMA (aOR = 1.95, a95%CI = 1.04–3.66, *p* = 0.038) (Table 5).

**TABLE 5:** Multiple logistic regression for factors correlated with the incidence of severe exacerbation during 6 months follow-up.

Characteristics (*N* = 695)	aOR	a95%IC	*p*-value
Inhalation therapy			
ICS/LABA/LAMA	Reference		
LAMA	0.53	0.23–1.23	0.138
LAMA/LABA	1.95	1.04–3.66	**0.038**
ICS/LABA	0.63	0.21–1.89	0.408
Others	0.76	0.25–0.31	0.631

**Note:** Factors in the logistic model: sex, age, treatment status at baseline, exacerbation in the past 1 year, severe exacerbation in the past 1 year, CAT score, mMRC score, Gold stage, group B/D, inhalation therapy; the bold *p*-values indicate statistical significance.

**Abbreviations:** CAT, COPD assessment test; mMRC, modified medical research council dyspnea scale; LABA, long-acting β2-agonist; LAMA, long-acting muscarinic antagonist; ICS, inhaled corticosteroid; aOR, adjusted odds ratio; a95% CI, adjusted 95% confidence interval.

## Discussion

To the best of our knowledge, this is the first real-world study to assess the effectiveness of inhalation therapies including mono, dual combination and triple combination therapies for symptomatic COPD patients in China.

Our results show that the MCID response rate (CAT improved ≥2) in symptomatic COPD patients treated with inhalation therapy was nearly 50% and the inhalation treatment of COPD patients with LAMA/LABA or triple therapy had a higher MCID response rate than LAMA or ICS/LABA. The total MCID response rate is consistent with previous studies showing that 51% of patients treated with umeclidinium/vilanterol, umeclidinium, or salmeterol achieved a clinical important improvement at week 24 (Vogelmeier et al., 2021). The benefits of triple treatment compared with mono and dual therapy are obvious in prospective clinical studies. Lee et al. demonstrated better improvements in St George’s Respiratory Questionnaire (SGRQ) scores in patients on inhaled triple therapy (tiotropium plus budesonide/formoterol) compared with those on monotherapy (tiotropium) (Lee et al., 2016). In the IMPACT study, fluticasone furoate/umeclidinium/vilanterol (FF/UMEC/VI) single-inhaler triple therapy was associated with a better clinically meaningful improvement in SGRQ score (defined as a decrease ≥4 units from baseline) compared with ICS/LABA (FF/VI) (Lipson et al., 2018). In the EMAX study, UMEC/VI showed greater improvements in the proportion of CAT responders versus UMEC at week 12 and week 24 (Maltais et al., 2019). A network meta-analysis demonstrated that LABA/LAMA combinations were associated with a greater improvement in SGRQ scores and the Transitional Dyspnea Index (TDI) than monotherapy (Oba et al., 2016). Our study provides consistent evidence in the real world that confirms the benefits of dual bronchodilation on symptom improvement compared with mono-bronchodilator therapy in symptomatic patients with COPD.

An RCT showed that the improvement over time in the total score on the SGRQ was greater in the LABA/LAMA group than in the ICS/LABA group, which is consistent with our results (Wedzicha et al., 2016). We found no difference in the MCID response rate between LAMA/LABA and triple inhalation therapy for symptomatic COPD patients. However, we also had results inconsistent with the Germany DACCORD real-world observational study, in which the response rate of patients with a clinically relevant improvement (CAT score ≥2-unit change from baseline) was higher in patients receiving LAMA/LABA compared with triple therapy patients (62 vs. 47%, respectively; *p* < 0.001) (Buhl et al., 2018). Poverty, a high rate of smoking, and indoor biomass burning are traditionally considerable issues in Asia (Gordon et al., 2014; Kim et al., 2019). COPD phenotypes in Asia may be somewhat different from those in Western countries (Kim et al., 2019). We believe that this difference may be due to the heterogeneity of the region and the study participants.

Our logistic regression model showed that female patients had a higher incidence of MCID. In Asian cities, the characteristics of COPD patients vary and the history of exposure to biomass fuels is related to frequency of symptoms and severe airflow limitation (Oh et al., 2013). Our previous study showed that nearly 70% of female in COPD patients were exposed to biomass smoke exposure alone. It has also been demonstrated that COPD patients with biomass exposure alone have higher CAT scores than patients with only smoke or occupational exposure (Duan et al., 2020). These previous reports also show that female COPD patients have more severe symptoms. We consider that these factors lead to higher MCID, because patients with more severe symptoms are more likely to obtain a 2 units reduction in the CAT score. We also found that patients treated with ICS/LABA/LAMA or LABA/LAMA were more likely to attain MCID than patients treated with LAMA. We have discussed this before, so we will not repeat it here.

In our study, we chose the MCID, which was defined as attaining a CAT decrease ≥2 during the 6 months follow-up, as our main effectiveness indicator in patients treated with inhalation bronchodilators. In clinical practice, it is time-consuming and impractical to monitor several different patient-reported outcome (PRO) measures such as CAT, SGRQ, self-administered computerized-Transition Dyspnea Index (SAC-TDI), and Evaluating Respiratory Symptoms (E-RS) (Vogelmeier et al., 2021). Previous systematic reviews supported the reliability and validity of the CAT and concluded that the tool is responsive to interventions. Furthermore, the correlation between CAT and SGRQ scores is typically quite high (convergent validity using Pearson’s correlation coefficient: 0.69–0.82 and 0.63), which has also been demonstrated in a systematic review (Gupta et al., 2014). Moreover, a large variety of questionnaires brings many difficulties to clinical practice and popularization. We think two or more PRO measures are more suitable for RCTs. A single CAT score for assessing systemic symptoms is more operable in real-world clinical practice and has been used in previous studies (Buhl et al., 2018).

We found no significant differences in the incidence of acute exacerbations during the 6 months follow-up period between different inhalation therapies in symptomatic COPD patients. In the past, there has been controversy regarding the risk of acute exacerbations after treatment with different inhalation therapies (Wedzicha et al., 2016; Lipson et al., 2018; Papi et al., 2018; Maltais et al., 2019; Suissa et al., 2019; Wang et al., 2021). However, we found that there were certain differences in the incidence of hospitalization-related acute exacerbations during the 6-months follow-up period between different inhalation therapies in symptomatic COPD patients. We found that COPD patients treated with LAMA had a lower incidence of severe exacerbations than LABA/LAMA patients. A network meta-analysis showed that all LAMAs are equally effective in preventing moderate-to-severe exacerbations, but the concomitant use of LABA may not enhance the efficacy of LAMAs in preventing COPD exacerbations (Oba and Lone, 2015). The EMAX randomized trial conducted in low exacerbation risk patients with COPD not receiving ICS showed that there was no difference in the occurrence of severe exacerbations between the umeclidinium/vilanterol and umeclidinium treatment groups (Maltais et al., 2019). It is known that previous exacerbation history is a reliable predictor of future exacerbations (Singh et al., 2019; Singh et al., 2020). In our study, the LAMA group had a higher proportion of patients with a history of exacerbations during the previous year than patients treated with LABA/LAMA (65.7 vs. 52.3%, *p* = 0.016). We think that this difference in the history of acute exacerbation between the LAMA and LABA/LAMA groups may be the main reason for this result.

Furthermore, inhalation treatment of COPD patients with ICS/LABA presented a lower incidence of severe exacerbations than LABA/LAMA. In previous research, there has been controversy regarding the risk of severe exacerbations between different inhalation treatments. In a real-world clinical practice setting of COPD treatment, the hazard ratio (HR) of severe COPD exacerbations associated with LABA/LAMA relative to ICS/LABA was 0.94. This study showed that combined LABA/LAMA inhalers appear to be as effective as combined ICS/LABA inhalers in preventing COPD exacerbations (Suissa et al., 2019), but an RCT demonstrated that the time to the first severe exacerbation was longer in the LABA/LAMA group than in the ICS/LABA group (HR 0.81; 95% CI, 0.66 to 1.00; *p* = 0.046) (Wedzicha et al., 2016). Another RCT showed that the annual rate of severe exacerbations during treatment was 0.15 among those assigned to ICS/LABA and 0.19 among those assigned to LABA/LAMA (Lipson et al., 2018). We consider that this difference may be due to the heterogeneity of the study population and the history of severe exacerbations between LABA/LAMA and ICS/LABA groups in the previous year. In our study, we also found that patients treated with LABA/LAMA had a higher incidence of severe exacerbation than those on triple inhalation therapy, which was consistent with a previous study. A matched cohort of 1,647 patients with COPD in a UK primary care database found that triple therapy reduced the exacerbation risk (HR 0.87, 95% CI 0.76–0.99) compared with LAMA/LABA dual therapy (Voorham et al., 2019). In the IMPACT study, triple therapy resulted in a lower rate of hospitalization due to COPD than LABA/LAMA (rate ratio with triple therapy, 0.66; 95% CI, 0.56 to 0.78; 34% difference; *p* < 0.001), but the rate was not significantly lower with triple therapy than with ICS/LABA (rate ratio with triple therapy, 0.87; 95% CI, 0.76 to 1.01; 13% difference; *p* = 0.06), which is consistent with our study (Lipson et al., 2018). This trial also demonstrated that these benefits were observed regardless of the patients’ blood eosinophil levels at randomization. We think this difference may be related to the effect of ICS on exacerbation prevention (Singh et al., 2019). Finally, in the multivariate analysis, we showed that the incidence of severe exacerbations in patients receiving LABA/LAMA treatment was higher than that of patients on triple inhalation therapy, which indirectly reflects the differences in the rate of severe exacerbations in other treatment groups, which may be related to the history of exacerbation before treatment.

There are some limitations to this study. First, the study did not correct the Charlson comorbidity index due to the limitations of real-world studies, but we evaluated other chronic pulmonary diseases, concurrent malignancy, severe heart, liver, or kidney diseases based on actual diagnoses from case records, which may reduce the confounding deviation of comorbidities to a certain extent. Second, according to current COPD treatment guidelines, blood eosinophil counts should be taken into consideration when deciding whether to initiate ICS treatment in combination with a LABA and/or LAMA (Singh et al., 2019). Our study did not include blood eosinophils in the multivariate analysis, which could cause a certain selection bias. However, it is likely that blood eosinophil counts were not considered in the treatment decisions observed in the current study, since the study was conducted prior to the inclusion of this recommendation. We also excluded patients diagnosed with asthma in our study, which may reduce this bias. Third, our study may have a relatively low incidence of exacerbation due to the short follow-up time. In the future, we may need to further explore and carry out follow-up studies on the acute exacerbations of these patients. Additionally, we did not include COPD patients in the less symptomatic groups into the study due to the fact that there are fewer COPD patients in groups A and C (8.7%) in these 12 comprehensive hospitals (Supplementary Table S3). In the future, we may need to cooperate with community hospitals to further expand the number of patients in groups A and C to supplement real-world data. Finally, we did not discuss the impact of the different types of inhalers, which may have influenced the selection of medications based on patient preference. However, these patients received inhalation training at the patient health management office after receiving the inhaler at their first visit. Therefore, each of our participants was able to use the inhaler correctly after assessment and inhalation training.

## Conclusion

The incidence of MCID in symptomatic COPD patients treated with inhalation therapy was nearly 50%. Patients treated with LABA/LAMA or ICS/LABA/LAMA were more likely to attain MCID than patients treated with LAMA. Patients treated with LABA/LAMA had a higher incidence of severe exacerbations than patients given ICS/LABA/LAMA.

## Data Availability

The raw data supporting the conclusion of this article will be made available by the authors, without undue reservation.
