# Supplementary material for: Real-World Effectiveness of Inhalation Therapy Among Patients With Symptomatic COPD in China: A Multicenter Prospective Study
Source: Front Pharmacol. 2021 Sep 21;12:753653. doi: 10.3389/fphar.2021.753653 (PMC8490668; doi:10.3389/fphar.2021.753653)
Supplement: Supplementary file 1 [file Table1.docx]

Table S1 Hospitals participating in this study

| Number | Hospital (Province, Country) |
| --- | --- |
| 1 | The Second Xiangya Hospital of Central South University (Hunan, China) |
| 2 | Zhuzhou Central Hospital (Hunan, China) |
| 3 | Hunan Prevention and Treatment Institute for Occupational Diseases (Hunan, China) |
| 4 | The Third Hospital of Changsha (Hunan, China) |
| 5 | Affiliated Hospital of Guilin Medical University (Guangxi Zhuang Autonomous Region, China) |
| 6 | The First Affiliated People's Hospital of Shaoyang College (Hunan, China) |
| 7 | The Second People’s Hospital of Guilin (Guangxi Zhuang Autonomous Region, China) |
| 8 | The No.1 Traditional Chinese Medicine Hospital in Changde (Hunan, China) |
| 9 | The First People’s Hospital of Huaihua (Hunan, China) |
| 10 | Longshan County Hospital of Traditional Chinese Medicine (Xiangxi Autonomous Prefecture, China) |
| 11 | Central Hospital of Xiangtan (Hunan, China) |
| 12 | The Eighth Hospital of Changsha (Hunan, China) |
