# Supplementary material for: Real-World Effectiveness of Inhalation Therapy Among Patients With Symptomatic COPD in China: A Multicenter Prospective Study
Source: Front Pharmacol. 2021 Sep 21;12:753653. doi: 10.3389/fphar.2021.753653 (PMC8490668; doi:10.3389/fphar.2021.753653)
Supplement: Supplementary file 2 [file Table2.docx]

Table S2. Comparison of the exacerbation history in the past one year in patients treated with different main inhalation therapy

| Inhalation therapy | Total patients in different therapy, n | AE in the past  1 year, n(%) | No AE  in the past 1 year, n(%) | *P*-value | Severe AE in the past 1 year, n(%) | No severe AE in the past 1 year, n(%) | *P*-value | Frequency AE in the past 1 year, n(%) | No frequency AE in the past 1 year, n(%) | *P*-value |
| --- | --- | --- | --- | --- | --- | --- | --- | --- | --- | --- |
| LAMA vs LABA/LAMA |  |  |  | **0.016** |  |  | 0.187 |  |  | 0.921 |
| LAMA | 169 | 111(65.7) | 58(34.3) |  | 56(33.3) | 113(66.9) |  | 66(39.1) | 103(60.9) |  |
| LABA/LAMA | 149 | 78(52.3) | 71(47.7) |  | 60(40.3) | 89(59.7) |  | 59(39.6) | 90(60.4) |  |
| LAMA vs ICS/LABA |  |  |  | 0.378 |  |  | 0.090 |  |  | 0.401 |
| LAMA | 169 | 111(65.7) | 58(34.3) |  | 56(33.3) | 113(66.9) |  | 66(39.1) | 103(60.9) |  |
| ICS/LABA | 72 | 43(59.7) | 29(40.3) |  | 16(22.2) | 56(77.8) |  | 24(33.3) | 48(66.7) |  |
| LAMA vs ICS/LABA/LAMA |  |  |  | 0.133 |  |  | 0.315 |  |  | 0.912 |
| LAMA | 169 | 111(65.7) | 58(34.3) |  | 56(33.3) | 113(66.9) |  | 66(39.1) | 103(60.9) |  |
| ICS/LABA/LAMA | 245 | 143(58.4) | 102(41.6) |  | 93(38.0) | 152(62.0) |  | 97(39.6) | 148(60.4) |  |
| LABA/LAMA vs ICS/LABA |  |  |  | 0.302 |  |  | **0.008** |  |  | 0.367 |
| LABA/LAMA | 149 | 78(52.3) | 71(47.7) |  | 60(40.3) | 89(59.7) |  | 59(39.6) | 90(60.4) |  |
| ICS/LABA | 72 | 43(59.7) | 29(40.3) |  | 16(22.2) | 56(77.8) |  | 24(33.3) | 48(66.7) |  |
| ICS/LABA vs ICS/LABA/LAMA |  |  |  | 0.837 |  |  | **0.013** |  |  | 0.337 |
| ICS/LABA | 72 | 43(59.7) | 29(40.3) |  | 16(22.2) | 56(77.8) |  | 24(33.3) | 48(66.7) |  |
| ICS/LABA/LAMA | 245 | 143(58.4) | 102(41.6) |  | 93(38.0) | 152(62.0) |  | 97(39.6) | 148(60.4) |  |
| LABA/LAMA vs ICS/LABA/LAMA |  |  |  | 0.243 |  |  | 0.648 |  |  | 0.999 |
| LABA/LAMA | 169 | 78(52.3) | 71(47.7) |  | 60(40.3) | 89(59.7) |  | 59(39.6) | 90(60.4) |  |
| ICS/LABA/LAMA | 245 | 143(58.4) | 102(41.6) |  | 93(38.0) | 152(62.0) |  | 97(39.6) | 148(60.4) |  |

**Note:** For comparison, Chi-square or Fisher’s test was used for categorical variables; the bold *P*-values indicate statistical significance.

**Abbreviations**: AE, acute exacerbation; LABA, Long-acting β2-agonist; LAMA, Long-acting muscarinic antagonist; ICS, Inhaled corticosteroid.
