# Supplementary material for: Real-World Effectiveness of Inhalation Therapy Among Patients With Symptomatic COPD in China: A Multicenter Prospective Study
Source: Front Pharmacol. 2021 Sep 21;12:753653. doi: 10.3389/fphar.2021.753653 (PMC8490668; doi:10.3389/fphar.2021.753653)
Supplement: Supplementary file 3 [file Table3.docx]

**Table S3** Response rate of MCID during 6-month follow up between BD group and AC group in COPD patients (N=761)

|  | N | Patients with MCID, n(%) | Patients without MCID, n(%) | *P*-value |
| --- | --- | --- | --- | --- |
| ABCD group |  |  |  | **0.048** |
| A+C | 66 | 24(36.4) | 42(63.6) |  |
| B+D | 695 | 341(49.1) | 354(50.9) |  |

**Note:** For comparison, Chi-square was used for categorical variables; the bold *P*-values indicate statistical significance.

**Abbreviations**: MCID, minimum clinically important difference; ABCD group was classified using Global Initiative for Chronic Obstructive Lung Disease (GOLD) criteria.
